# Supplementary material for: Stacking Model‐Based Classifiers for Dealing With Multiple Sets of Noisy Labels
Source: Biom J. 2025 Mar 12;67(2):e70042. doi: 10.1002/bimj.70042 (PMC11898607; doi:10.1002/bimj.70042)
Supplement: Supplementary file 1 — Supporting Information [file BIMJ-67-e70042-s001.zip › density-based-ensemble-model-main/competitors_real_data/gpml-matlab-master/doc/index.html]

Documentation for GPML Matlab Code


# Documentation for GPML Matlab Code version 4.2

## 1) What?

The code provided here originally demonstrated the main algorithms
from Rasmussen and Williams: Gaussian Processes for
Machine Learning. It has since grown to allow more likelihood
functions, further inference methods and a flexible framework for
specifying GPs.
Other GP packages can be found here.

The code is written by Carl Edward Rasmussen and Hannes Nickisch; it runs on
both Octave 3.2.x
and Matlab® 7.x and later.
The code is based on previous versions
written by Carl Edward Rasmussen and Chris Williams.

## 2) Download, Install and Documentation

All the code including demonstrations and html documentation can be
downloaded from
gitlab
or in a tar
or zip
archive file.

Minor changes and incremental bugfixes to the current version are
documented in the changelog, changes from
previous versions are documented
in README.

Please read the copyright notice.

After unpacking the tar or zip file you will find 7 subdirectories:
cov, doc, inf, lik, mean, prior and util. It is not necessary to install
anything to get started, just run the startup script to set your path.

Details about the directory contents and on how to compile mex
files can be found in the README. The getting
started guide is the remainder of the html file you are currently
reading (also available at http://gaussianprocess.org/gpml/code/matlab/doc).
A Developer's Guide containing technical documentation is
found in manual.pdf, but for the casual user,
the guide is below.

## 3) Getting Started

Gaussian Processes (GPs) can conveniently be used for Bayesian
supervised learning, such as regression and classification. In its
simplest form, GP inference can be implemented in a few lines of
code. However, in practice, things typically get a little more
complicated: you might want to use complicated covariance functions
and mean functions, learn good values for hyperparameters, use
non-Gaussian likelihood functions (rendering exact inference
intractable), use approximate inference algorithms, or combinations of
many or all of the above. This is what the GPML software package
does.

The remainder of section 3 first presents some essential components of
the software in the next subsection. This enables understand the usage
for the gp function described in section 3b). A practical
example follows in 3c), and the section concludes with a more detailed
overview in 3d).

### 3a) Some Essential Components

Before going straight to the examples, just a brief note about the
organization of the package. There are four essential types of objects which you
need to know about:

**Gaussian Process**: A Gaussian Process is fully specified by a *mean function* and a *covariance function*. These functions are specified separately, and consist of a specification of a functional form as well as a set of parameters called *hyperparameters*, see below. **Mean functions**: Several mean functions are available, an overview is provided by the meanFunctions help function (type help meanFunctions to get help), and an example is the meanLinear function. **Covariance functions**: Several covariance functions are available, an overview is provided by the covFunctions help function (type help covFunctions to get help), and an example is the covSEard "Squared Exponential with Automatic Relevance Determination" covariance function. For a more comprehensive overview of mean and covariance functions, see section 3d) below. **Hyperparameters**: GPs are specified using mean and covariance functions which typically have free parameters called *hyperparameters*. Also likelihood functions may have such parameters. Hyperparameters are represented in a struct with the fields mean, cov and lik (some of which may be empty). It is important that the number of elements in each of the fields in the hyperparameter struct matches the specification of the mean, covariance and likelihood functions. **Likelihood Functions**: The likelihood function specifies the probability of the observations given the GP (and the hyperparameters). Several likelihood functions are available, an overview is provided by the likFunctions help function (type help likFunctions to get help). Example likelihood functions include likGauss the Gaussian likelihood for regression and likLogistic the logistic likelihood for classification. **Inference Methods**: The inference methods specify how to compute with the model, i.e. how to infer the (approximate) posterior process, how to find hyperparameters, evaluate the log marginal likelihood and how to make predictions. Several inference methods are avaiable, and overview is provided by the infMethods help file (type help infMethods to get help). An example inference method is infGaussLik for exact inference (regression with Gaussian likelihood). **Covariance approximations**: The covariance approximation as used by apx determines an important computational aspect in GP inference: the scalability with the number of inputs. We offer four options here: a) Exact covariance computations serving as default option (demoRegression, demoClassification), b) sparse covariance approximations exploiting inducing points and conditional independence (apxSparse, demoSparse), c) grid-based covariance approximations using interpolation from a rectilinear grid of inducing points and exploiting the resulting Kronecker and Toeplitz property of covariance matrices (apxGrid, demoGrid1d, demoGrid2d), and d) state space covariance approximations exploiting a state space representation of univariate GPs (apxState, demoState).

### 3b) The gp Function

Using the GPML package is simple, there is only one single function to
call: gp, it does posterior inference, learns
hyperparameters, computes the marginal likelihood and makes
predictions. Generally, the gp function takes the following arguments:
a hyperparameter struct, an inference method, a mean function, a
covariance function, a likelihood function, training inputs, training
targets, and possibly test cases. The exact computations done by the
function is controlled by the number of input and output arguments in
the call. Here is part of the help message for the gp function (follow the link to see the whole thing):

```
  function [varargout] = gp(hyp, inf, mean, cov, lik, x, y, xs, ys)

  [ ... snip ... ]

  Two modes are possible: training or prediction: if no test cases are
  supplied, then the negative log marginal likelihood and its partial
  derivatives wrt the hyperparameters is computed; this mode is used to fit the
  hyperparameters. If test cases are given, then the test set predictive
  probabilities are returned. Usage:

    training: [nlZ dnlZ          ] = gp(hyp, inf, mean, cov, lik, x, y);
  prediction: [ymu ys2 fmu fs2   ] = gp(hyp, inf, mean, cov, lik, x, y, xs);
          or: [ymu ys2 fmu fs2 lp] = gp(hyp, inf, mean, cov, lik, x, y, xs, ys);

  [ ... snip ... ]
```

Here x and y are training inputs and outputs, and
xs and ys are test set inputs and outputs,
nlZ is the negative log marginal likelihood and
dnlZ its partial derivatives wrt the hyperparameters (which
are used for training the hyperparameters). The prediction outputs are
ymu and ys2 for test output mean and covariance, and
fmu and fs2 are the equivalent quenteties for the
corresponding latent variables. Finally, lp are the test
output log probabilities.

### 3c) A Practical Example

To get started, let's consider the simple example of one-dimensional
non-linear regression on data corrupted by Gaussian noise. Our data set is

```
  x = gpml_randn(0.8, 20, 1);                 % 20 training inputs
  y = sin(3*x) + 0.1*gpml_randn(0.9, 20, 1);  % 20 noisy training targets
  xs = linspace(-3, 3, 61)';                  % 61 test inputs
```

We need specify the mean, covariance and likelihood functions

```
  meanfunc = [];                    % empty: don't use a mean function
  covfunc = @covSEiso;              % Squared Exponental covariance function
  likfunc = @likGauss;              % Gaussian likelihood
```

Finally we initialize the hyperparameter struct

```
  hyp = struct('mean', [], 'cov', [0 0], 'lik', -1);
```

Note, that the hyperparameter struct must have the three fields
mean, cov and lik. Each field must have the
number of elements which corresponds to the functions specified. In
our case, the mean function is empty, so takes no parameters. The
covariance function is covSEiso, the squared exponential with
isotropic distance measure, which takes two parameters
(see help covSEiso). As explained in the help for the
function, the meaning of the hyperparameters is "log of the
length-scale" and the "log of the signal std dev". Initializing both of
these to zero, corresponds to length-scale and signal std dev to be
initialized to one. The Gaussian likelihood function has a single
parameter, which is the log of the noise standard deviation, setting
the log to zero corresponds to a standard deviation of exp(-1)=0.37.

A common situation with modeling with GPs is that approprate
settings of the hyperparameters are not known a priori. The situation
is reflected in the above initialization of the hyperparameters, where
the values values are specified without careful justification, perhaps
based on some vague notions of the magnitudes likely to be
involved. Thus, a common task is to set hyperparameters by optimizing
the (log) marginal likelihood. This is done as follows

```
  hyp2 = minimize(hyp, @gp, -100, @infGaussLik, meanfunc, covfunc, likfunc, x, y);
```

The minimize function minimizes the negative log marginal
likelihood, which is returned by the gp function, together
with the partial derivatives wrt the hyperparameters. The inference
method is specified to be infGaussLik exact inference. The
minimize function is allowed a computational budget of 100 function
evaluations. The hyperparameters found are

```
  hyp2 =
    mean: []
     cov: [-0.6352 -0.1045]
     lik: -2.3824
```

showing that the covariance characteristic length-scale is
exp(-0.6352)=0.53, the signal std dev is
exp(-0.1045)=0.90 and the noise std dev is
exp(-2.3824)=0.092 in good agreement with the data
generating process.

To make predictions using these hyperparameters

```
  [mu s2] = gp(hyp2, @infGaussLik, meanfunc, covfunc, likfunc, x, y, xs);
```

To plot the predictive mean at the test points together with the
predictive 95% confidence bounds and the training data

```
  f = [mu+2*sqrt(s2); flipdim(mu-2*sqrt(s2),1)];
  fill([xs; flipdim(xs,1)], f, [7 7 7]/8)
  hold on; plot(xs, mu); plot(x, y, '+')
```

which produces a plot like this
  

### 3d) A More Detailed Overview

The previous section shows a minimalist example, using the central
concepts of GPML. This section provides a less simplistic overview,
mainly through a number of useful comments and pointers to more
complete treatments.

In order to be able to find things, the toolbox is organized into the
following directories mean for mean functions, cov
for covariance functions, lik for likelihood functions,
inf for inference methods prior for priors and
mcmc for Markov Chain monte Carlo tools, doc for
documentation and util for general utilities.

In addition to this structure, the naming of functions within some of
these directories also start with the letters mean,
cov, lik and inf as a further mnemonic aid.

The following paragraphs contain useful further details about some of
the concepts we have already used.

**Mean functions** and **covariance functions**. As detailed in
meanFunctions and
covFunctions there are
actually two types of these, simple and composite. Composite
functions are used to compose simple functions into more
expressive structures. For example

```
  meanfunc = {@meanSum, {@meanLinear, @meanConst}};
```

specifies a mean function which is a sum of a linear and a constant
part. Note how composite functions are specified using cell
arrays. Note also, that the corresponding mean hyperparameter will
consist of a vector containing the concatenation of the different
parts of the mean function. If you call a mean or covariance function
without arguments, they will return a string indicating the number of
hyperparameters expected; this also works for composite covariance
functions; the letter D in the string designates the
dimension of the inputs.

**Likelihood functions**. As detailed in likFunctions there are also
simple and composite likelihood functions; the only composite
likelihood function is likMix,
which implements a mixture of multiple likelihoods.

**Inference methods**.

Whereas all mean functions and covariance functions may be used in
any context, there are some restrictions on which likelihood functions
may be used with which inference method. An exhaustive compatibility
matrix between likelihoods (rows) and inference methods (columns) is
given in the table below:

|  |  |  |  |  |  |  |  |  |  |  |
| --- | --- | --- | --- | --- | --- | --- | --- | --- | --- | --- |
| Plain Regression Likelihoods | | | | | | | | | | |
|  | Cov. approxim.: dense, sparse, grid or state |  |  |  | ()\* |  |  |  | type, output domain | alternative names |
| Inference | GPML name | Gaussian noise  infGaussLik | Laplace  infLaplace | Variational Bayes  infVB | EP  infEP | Kullback Leibler  infKL | Sampling  infMCMC | LOO  infLOO |  |  |
| Likelihood |  |  |  |  |  |  |  |  |  |  |
| Gaussian | likGauss |  |  |  |  |  |  |  | regression, IR |  |
| Warped Gaussian | likGaussWarp |  |  |  |  |  |  |  | regression, IR |  |
| Gumbel | likGumbel |  |  |  |  |  |  |  | regression, IR | extremal value regression |
| Sech squared | likSech2 |  |  |  |  |  |  |  | regression, IR | logistic distribution |
| Laplacian | likLaplace |  |  |  |  |  |  |  | regression, IR | double exponential |
| Student's t | likT |  |  |  |  |  |  |  | regression, IR |  |
| Classification Likelihoods | | | | | | | | | | |
| Uniform | likUni |  |  |  |  |  |  |  | classification, ±1 | label noise |
| Error function | likErf |  |  |  |  |  |  |  | classification, ±1 | probit regression |
| Logistic function | likLogistic |  |  |  |  |  |  |  | classification, ±1 | logistic regression  logit regression |
| Generalized Linear Model Likelihoods | | | | | | | | | | |
|  | Cov. approxim.: dense, sparse, grid or state |  |  |  | ()\* |  |  |  | type, output domain | alternative names |
| Inference | GPML name | Gaussian noise  infGaussLik | Laplace  infLaplace | Variational Bayes  infVB | EP  infEP | Kullback Leibler  infKL | Sampling  infMCMC | LOO  infLOO |  |  |
| Likelihood |  |  |  |  |  |  |  |  |  |  |
| Weibull | likWeibull |  |  |  |  |  |  |  | positive data, IR+\{0} | nonnegative regression |
| Gamma | likGamma |  |  |  |  |  |  |  | positive data, IR+\{0} | nonnegative regression |
| Exponential | likExp |  |  |  |  |  |  |  | positive data, IR+\{0} | nonnegative regression |
| Inverse Gaussian | likInvGauss |  |  |  |  |  |  |  | positive data, IR+\{0} | nonnegative regression |
| Poisson | likPoisson |  |  |  | ()\*\* |  |  |  | count data, IN | Poisson regression |
| Negative Binomial | likNegBinom |  |  |  |  |  |  |  | count data, IN | negative binomial regression |
| Beta | likBeta |  |  |  |  |  |  |  | interval data, [0,1] | range regression |
| Composite Likelihoods | | | | | | | | | | |
| Mixture | likMix |  |  |  |  |  |  |  | classification, ±1 and regression, IR | mixing meta likelihood |

\* EP supports FITC via a separate function. No support for the generic covariance
approximations.   
\*\* EP might not converge in some cases since quadrature is used.

All of the objects described above are written in a modular way, so
you can add functionality if you feel constrained despite the
considerable flexibility provided. Details about how to do this are provided
in the developer documentation.

Inference by MCMC sampling is the only inference method that cannot be
used as a black box. Also gradient-based marginal likelihood optimisation
is not possible with MCMC. Please see usageSampling
for a toy example illustrating the usage of the implemented samplers.

## 4) Practice

Instead of exhaustively explaining all the possibilities, we
will give two illustrative examples to give you the idea; one for
regression and one for classification. You can either follow the
example here on this page, or using the two scripts demoRegression and demoClassification (using the
scripts, you still need to follow the explanation on this page).

### 4a) Simple Regression

You can either follow the example here on this page, or use the script demoRegression.

This is a simple example, where we first generate n=20
data points from a GP, where the inputs are scalar (so that it is easy
to plot what is going on). We then use various other GPs to make
inferences about the underlying function.

First, generate some data from a Gaussian process (it is not essential to
understand the details of this):

```
  clear all, close all
 
  meanfunc = {@meanSum, {@meanLinear, @meanConst}}; hyp.mean = [0.5; 1];
  covfunc = {@covMaterniso, 3}; ell = 1/4; sf = 1; hyp.cov = log([ell; sf]);
  likfunc = @likGauss; sn = 0.1; hyp.lik = log(sn);
 
  n = 20;
  x = gpml_randn(0.3, n, 1);
  K = feval(covfunc{:}, hyp.cov, x);
  mu = feval(meanfunc{:}, hyp.mean, x);
  y = chol(K)'*gpml_randn(0.15, n, 1) + mu + exp(hyp.lik)*gpml_randn(0.2, n, 1);

  plot(x, y, '+')
```

Above, we first specify the mean function meanfunc,
covariance function covfunc of a GP and a likelihood
function, likfunc. The corresponding hyperparameters are
specified in the hyp structure:

The **mean function** is composite, adding (using meanSum
function) a linear (meanLinear) and a constant
(meanConst) to get an affine function. Note, how the
different components are composed using cell arrays. The hyperparameters
for the mean are given in hyp.mean and consists of a single
(because the input will one dimensional, i.e. D=1) slope (set
to 0.5) and an off-set (set to 1). The number and the order of these
hyperparameters conform to the mean function specification. You can
find out how many hyperparameters a mean (or covariance or likelihood
function) expects by calling it without arguments, such as
feval(meanfunc{:}). For more information on mean functions
see meanFunctions and the directory
mean/.

The **covariance function** is of the Matérn form with isotropic
distance measure covMaterniso. This covariance function is
also composite, as it takes a constant (related to the smoothness of
the GP), which in this case is set to 3. The covariance
function takes two
hyperparameters, a characteristic length-scale ell and the
standard deviation of the signal sf. Note, that these
positive parameters are represented in hyp.cov using their
logarithms. For more
information on covariance functions see covFunctions and cov/.

Finally, the **likelihood function** is specified to be
Gaussian. The standard deviation of the noise sn is set to
0.1. Again, the representation in the hyp.lik is given in
terms of its logarithm. For more information about likelihood
functions, see likFunctions and lik/.

Then, we generate a dataset with n=20 examples. The inputs
x are drawn from a unit Gaussian (using the
gpml\_randn utility, which generates unit Gaussian pseudo
random numbers with a specified seed). We then evaluate the covariance
matrix K and the mean vector m by calling the
corresponding functions with the hyperparameters and the input
locations x. Finally, the targets y are computed by
drawing randomly from a Gaussian with the desired covariance and mean
and adding Gaussian noise with standard deviation
exp(hyp.lik). The above code is a bit special because we
explicitly call the mean and covariance functions (in order to
generate samples from a GP); ordinarily, we would only directly call
the gp function.

  

Let's ask the model to compute the (joint) negative log probability
(density) nlml (also called marginal likelihood or evidence)
and to generalize from the training data to other (test) inputs
z:

```
  nlml = gp(hyp, @infGaussLik, meanfunc, covfunc, likfunc, x, y)

  z = linspace(-1.9, 1.9, 101)';
  [m s2] = gp(hyp, @infGaussLik, meanfunc, covfunc, likfunc, x, y, z);

  f = [m+2*sqrt(s2); flipdim(m-2*sqrt(s2),1)]; 
  fill([z; flipdim(z,1)], f, [7 7 7]/8)
  hold on; plot(z, m); plot(x, y, '+')
```

The gp function is called with a struct of hyperparameters
hyp, and inference method, in this case @infGaussLik for exact inference and the
mean, covariance and likelihood functions, as well as the inputs and
outputs of the training data. With no test inputs, gp returns
the negative log probability of the training data, in this example
nlml=11.97.

To compute the predictions at test locations we add the test inputs
z as a final argument, and gp returns the mean
m variance s2 at the test location. The program is
using algorithm 2.1 from the GPML book. Plotting the
mean function plus/minus two standard deviations (corresponding to a
95% confidence interval):

  

Typically, we would not a priori know the values of the
hyperparameters hyp, let alone the form of the mean,
covariance or likelihood functions. So, let's pretend we didn't know
any of this. We assume a particular structure and learn suitable
hyperparameters:

```
  covfunc = @covSEiso; hyp2.cov = [0; 0]; hyp2.lik = log(0.1);

  hyp2 = minimize(hyp2, @gp, -100, @infGaussLik, [], covfunc, likfunc, x, y);
  exp(hyp2.lik)
  nlml2 = gp(hyp2, @infGaussLik, [], covfunc, likfunc, x, y)

  [m s2] = gp(hyp2, @infGaussLik, [], covfunc, likfunc, x, y, z);
  f = [m+2*sqrt(s2); flipdim(m-2*sqrt(s2),1)];
  fill([z; flipdim(z,1)], f, [7 7 7]/8)
  hold on; plot(z, m); plot(x, y, '+')
```

First, we guess that a squared
exponential covariance function covSEiso may be suitable.
This covariance function takes two hyperparameters: a characteristic
length-scale and a signal standard deviation (magnitude). These
hyperparameters are non-negative and represented by their logarithms;
thus, initializing hyp2.cov to zero, correspond to unit
characteristic length-scale and unit signal standard deviation.
The likelihood hyperparameter in hyp2.lik is also
initialized. We assume that the mean function is zero, so we simply
ignore it (and when in the following we call gp,
we give an empty argument for the mean function).

In the following line, we optimize over the hyperparameters, by
minimizing the negative log marginal likelihood w.r.t. the
hyperparameters. The third parameter in the call to minimize limits the number of function
evaluations to a maximum of 100. The inferred noise standard deviation is
exp(hyp2.lik)=0.15, somewhat larger than the one used to
generate the data (0.1). The final negative log marginal likelihood is
nlml2=14.13, showing that the joint probability (density) of
the training data is about exp(14.13-11.97)=8.7 times smaller
than for the setup actually generating the data. Finally, we plot the
predictive distribution.

  

This plot shows clearly, that the model is indeed quite different from
the generating process. This is due to the different specifications of
both the mean and covariance functions. Below we'll try to do a better
job, by allowing more flexibility in the specification.

Note that the confidence interval in this plot is the confidence for
the distribution of the (noisy) *data*. If instead you want the
confidence region for the underlying *function*, you should
use the 3rd and 4th output arguments from gp as
these refer to the latent process, rather than the data points.

```
  hyp.cov = [0; 0]; hyp.mean = [0; 0]; hyp.lik = log(0.1);
  hyp = minimize(hyp, @gp, -100, @infGaussLik, meanfunc, covfunc, likfunc, x, y);
  [m s2] = gp(hyp, @infGaussLik, meanfunc, covfunc, likfunc, x, y, z);
 
  f = [m+2*sqrt(s2); flipdim(m-2*sqrt(s2),1)];
  fill([z; flipdim(z,1)], f, [7 7 7]/8)
  hold on; plot(z, m); plot(x, y, '+');
```

Here, we have changed the specification by adding the affine mean
function. All the hyperparameters are learnt by optimizing the
marginal likelihood.

  

This shows that a much better fit is achieved when allowing a mean
function (although the covariance function is still different from
that of the generating process).

#### Guiding marginal likelihood optimisation with a hyperprior

It can be usefull to put a prior distribution on (a part of)
the hyperparameters. Sometimes, one may want to exclude some hyperparameters
from the optimisation i.e. fix their values beforehand and treat them as constants.

In these cases, a hyperprior comes to bear. A hyperprior is specified by
augmenting the inf parameter of gp.m In the regression
before, we had inf = @infGaussLik;. To put a Gaussian prior
on the first mean hyperparameter hyp.mean(1) and a Laplacian prior
on the second mean hyperparameter hyp.mean(2) and wished to fix
the noise variance hyperparameter hyp.lik, we simple need to
set up the corresponding prior structure as detailed below.

```
  mu = 1.0; s2 = 0.01^2;
  prior.mean = {{@priorGauss,mu,s2}; {'priorLaplace',mu,s2}};
  prior.lik = {{@priorDelta}};
  inf = {@infPrior,@infGaussLik,prior};
  hyp = minimize(hyp, @gp, -100, inf, meanfunc, covfunc, likfunc, x, y);
```

Further examples are provided in usagePrior.

### 4b) Sparse Approximate Regression based on inducing inputs

In case the number of training inputs x exceeds a few
thousands, exact inference takes too long. We offer the sparse
approximations to deal with these cases. The general idea is to use
inducing points u and to base the computations on
cross-covariances between training, test and inducing points only.
See demoSparse for a quick overview over
possible options.

Using sparse approximations is very simple, we just have to wrap
the covariance function covfunc into
apxSparse.m and call
gp.m with the inference method
infGaussLik.m as demonstrated by
the following lines of code.

```
  nu = fix(n/2); u = linspace(-1.3,1.3,nu)';
  covfuncF = {@apxSparse, {covfunc}, u};
  inf = @(varargin) infGaussLik(varargin{:}, struct('s', 0.0));
  [mF s2F] = gp(hyp, inf, meanfunc, covfuncF, likfunc, x, y, z);
```

In this code, a sparse approximate covariance function is defined by
composing the apxSparse function with a covariance function
and a set of inducing inputs. Further, an inference method
inf is defined by concatenating the struct('s', 0.0)
to the infGaussLik inference method. The value of the
appended s can be used to chose between s=1 for
Fully Independent Training Conditional (FITC) approximation, or
s=0 corresponding the Variational Free Energy (VFE)
approximation, or intermediate values of 0<s<1 corresponding
to Sparse Power Expectation Propagation (SPEP).

We define equispaced inducing points u that are shown in the
figure as black circles. Note that the predictive variance is overestimated
outside the support of the inducing inputs.
In a multivariate example where densely sampled inducing inputs are infeasible,
one can simply use a random subset of the training points.

```
  nu = fix(n/2); iu = randperm(n); iu = iu(1:nu); u = x(iu,:);
```

  

Another possibility is to specify the inducing inputs as a part of
the hyperparmeters using the field hyp.xu

```
  hyp.xu = u;
```

in which case optimization of the hyperparameters will also optimize
the inducing inputs (note that, when inducing inputs are given as a
field in the hyperparameters, then these take precedence over inducing
inputs specified in to the apxSparse function).

### 4c) Large scale regression exploiting grid structure

A covariance function factorising over coordinate axes evaluated on a
rectilinear (not necessarily equispaced) grid of data points leads
to a covariance matrix with Kronecker structure. This can be exploited
to scale GPs beyond the O(n³) limit. Observations not located on the grid
can be interpolated from the grid values. The example below
contains the most relevant code from the script
demoGrid2d, where we extrapolate a pixel image
beyond its boundaries. An instructive example in 1d can be found in
demoGrid1d.

For a comprehensive set of examples and more resources, see a  website by Andrew Wilson.

We start off by setting up the training data and the GP on a [-2,2]x[-3,3] lattice with 15600
pixels -- a size where a usual dense GP would be computationally infeasible. We use a lattice only
for the purpose of visualisation.

```
  x1 = linspace(-2,2,120); x2 = linspace(-3,3,130);
  x = apxGrid('expand',{x1',x2'});
  y = sin(x(:,2)) + x(:,1) + 0.1*gpml_randn(1,[size(x,1),1]);
```

Then, we define the locations where we would like to have the predictions i.e. a [-4,4]x[-6,6] lattice to see the result of the extrapolation.

```
  xs1 = linspace(-4,4,100); xs2 = linspace(-6,6,110);
  xs = apxGrid('expand',{xs1',xs2'});
```

Next, we construct the grid for KISS/SKI GP using an auxiliary function so that both training data x and the test data xs are properly covered.

```
  xg = apxGrid('create',[x;xs],true,[50,70]);
```

Now that the data set is well-defined, we specify our GP model along with initial values for the hyperparameter
by wrapping the covariance functions into apxGrid, GPML's grid-based covariance
approximation method.

```
  cov = {{@covSEiso},{@covSEiso}}; covg = {@apxGrid,cov,xg};
  mean = {@meanZero}; lik = {@likGauss};
  hyp.cov = zeros(4,1); hyp.mean = []; hyp.lik = log(0.1);
```

As a next step, we perform hyperparameter optimisation as with a usual GP model using the
inference method infGaussLik which only supports likGauss. In order to do so,
we specify parameters for the LCG: the maximum number of iterations and the convergence threshold
via the opt variable.

```
  opt.cg_maxit = 200; opt.cg_tol = 5e-3;
  infg = @(varargin) infGaussLik(varargin{:},opt);
  hyp = minimize(hyp,@gp,-50,infg,[],covg,[],x,y);
```

Finally, we make use of grid interpolation to compute predictions very rapidly with the
post.predict utility provided by the infGrid method.

```
  [post,nlZ,dnlZ] = infGrid(hyp,{@meanZero},covg,{@likGauss},x,y,opt);
  [fm,fs2,ym,ys2] = post.predict(xs);
```

Alternatively, we can use the usual pathway based on gp,

```
  post.L = @(a) zeros(size(a));
  ym = gp(hyp,infg,[],covg,[],x,post,xs);
```

where we have switched of the predictive variance computations as they are very demanding to
evaluate on an entire lattice. If the are required for a larger set of test points xs,
one can resort to sampling-based estimates.

The figure below summarizes what we have done. On the left, we see the training data and on the
right the GP predictive mean.

### 4d) Exercises for the reader

**Inference Methods**: Try using Expectation Propagation instead of exact inference in the above, by exchanging @infGaussLik with @infEP. You get exactly identical results, why? **Mean or Covariance**: Try training a GP where the affine part of the function is captured by the *covariance function* instead of the *mean function*. That is, use a GP with no explicit mean function, but further additive contributions to the covariance. How would you expect the marginal likelihood to compare to the previous case?

### 4e) Classification

You can either follow the example here on this page, or use the script demoClassification.

The difference between regression and classification isn't of
fundamental nature. We can use a Gaussian process latent function in
essentially the same way, it is just that the Gaussian likelihood
function often used for regression is inappropriate for
classification. And since exact inference is only possible for
Gaussian likelihood, we also need an alternative, approximate,
inference method.

Here, we will demonstrate binary classification, using two partially
overlapping Gaussian sources of data in two dimensions. First we
generate the data:

```
  clear all, close all
 
  n1 = 80; n2 = 40;                   % number of data points from each class
  S1 = eye(2); S2 = [1 0.95; 0.95 1];           % the two covariance matrices
  m1 = [0.75; 0]; m2 = [-0.75; 0];                            % the two means
 
  x1 = bsxfun(@plus, chol(S1)'*gpml_randn(0.2, 2, n1), m1);
  x2 = bsxfun(@plus, chol(S2)'*gpml_randn(0.3, 2, n2), m2);
 
  x = [x1 x2]'; y = [-ones(1,n1) ones(1,n2)]';
  plot(x1(1,:), x1(2,:), 'b+'); hold on;
  plot(x2(1,:), x2(2,:), 'r+');
```

120 data points are generated from two Gaussians with different
means and covariances. One Gaussian is isotropic and contains 2/3 of the data (blue), the
other is highly correlated and contains 1/3 of the points (red). Note,
that the labels for the targets are ±1 (and **not** 0/1).

In the plot, we superimpose the data points with the posterior
equi-probability contour lines for the probability of class two given
complete information about the generating mechanism

```
  [t1 t2] = meshgrid(-4:0.1:4,-4:0.1:4);
  t = [t1(:) t2(:)]; n = length(t);                 % these are the test inputs
  tmm = bsxfun(@minus, t, m1');
  p1 = n1*exp(-sum(tmm*inv(S1).*tmm/2,2))/sqrt(det(S1));
  tmm = bsxfun(@minus, t, m2');
  p2 = n2*exp(-sum(tmm*inv(S2).*tmm/2,2))/sqrt(det(S2));
  contour(t1, t2, reshape(p2./(p1+p2), size(t1)), [0.1:0.1:0.9]);
```

  

We specify a Gaussian process model as follows: a constant mean
function, with initial parameter set to 0, a squared exponential with
automatic relevance determination (ARD) covariance function covSEard. This covariance function has
one characteristic length-scale parameter for each dimension of the
input space, and a signal magnitude parameter, for a total of 3
parameters (as the input dimension is D=2). ARD with separate
length-scales for each input dimension is a very powerful tool to
learn which inputs are important for predictions: if length-scales are
short, inputs are very important, and when they grow very long
(compared to the spread of the data), the corresponding inputs will be
largely ignored. Both length-scales and the signal magnitude are
initialized to 1 (and represented in the log space). Finally, the
likelihood function likErf has the shape
of the error-function (or cumulative Gaussian), which doesn't take any
hyperparameters (so hyp.lik does not exist).

```
  meanfunc = @meanConst; hyp.mean = 0;
  covfunc = @covSEard; ell = 1.0; sf = 1.0; hyp.cov = log([ell ell sf]);
  likfunc = @likErf;

  hyp = minimize(hyp, @gp, -40, @infEP, meanfunc, covfunc, likfunc, x, y);
  [a b c d lp] = gp(hyp, @infEP, meanfunc, covfunc, likfunc, x, y, t, ones(n, 1));

  plot(x1(1,:), x1(2,:), 'b+'); hold on; plot(x2(1,:), x2(2,:), 'r+')
  contour(t1, t2, reshape(exp(lp), size(t1)), [0.1:0.1:0.9]);
```

We train the hyperparameters using minimize,
to minimize the negative log
marginal likelihood. We allow for 40 function evaluations,
and specify that inference should be done with the Expectation
Propagation (EP) inference method @infEP,
and pass the usual parameters. Training is done using algorithm 3.5
and 5.2 from the gpml
book. When computing test probabilities, we call gp
with additional test inputs, and as the last argument a vector of
targets for which the log probabilities lp should be
computed. The fist four output arguments of the function are mean and
variance for the targets and corresponding latent variables
respectively. The test set predictions are computed using algorithm
3.6 from the GPML
book. The contour plot for the predictive distribution is
shown below. Note, that the predictive probability is fairly close to the
probabilities of the generating process in regions of high data
density. Note also, that as you move away from the data, the
probability approaches 1/3, the overall class probability.

  

Examining the two ARD characteristic length-scale parameters after
learning, you will find that they are fairly similar, reflecting the
fact that for this data set, both inputs important.

#### Large scale classification using the FITC approximation

In case the number of training inputs x exceeds a few
hundreds, approximate inference using
infLaplace.m, infEP.m
and infVB.m
takes too long. As in regression, we offer the FITC approximation based on a low-rank
plus diagonal approximation to the exact covariance to deal with these
cases. The general idea is to use inducing points u and to
base the computations on cross-covariances between training, test and
inducing points only.

Using the FITC approximation is very simple, we just have to wrap the covariance
function covfunc into apxSparse.m
and call gp.m with the inference methods infLaplace.m
or infVB.m as demonstrated by the following
lines of code.

```
  [u1,u2] = meshgrid(linspace(-2,2,5)); u = [u1(:),u2(:)]; clear u1; clear u2
  nu = size(u,1);
  covfuncF = {@apxSparse, {covfunc}, u};
  inffunc = @infLaplace;                                                 % or infEP
  hyp = minimize(hyp, @gp, -40, inffunc, meanfunc, covfuncF, likfunc, x, y);
  [a b c d lp] = gp(hyp, inffunc, meanfunc, covfuncF, likfunc, x, y, t, ones(n,1));
```

We define equispaced inducing points u that are shown in the
figure as black circles. Alternatively, a random subset of the training
points can be used as inducing points.

  

#### Large scale classification exploiting the grid structure

Similar to regression using infGaussLik, we can perform approximate inference
using infLaplace to scale GPs beyond the O(n³) limit.
Please visit the  website by Seth Flaxman for an extended example and related datasets.

#### Exercise for the reader

**Inference Methods**: Use the Laplace Approximation for inference @infLaplace, and compare the approximate marginal likelihood for the two methods. Which approximation is best? **Covariance Function**: Try using the squared exponential with isotropic distance measure covSEiso instead of ARD distance measure covSEard. Which is best?

## 5) Acknowledgements

Innumerable colleagues have helped to improve this software. Some
of these are: John Cunningham, Máté Lengyel, Joris Mooij,
Iain Murray, David Duvenaud, Andrew McHutchon, Rowan McAllister, Daniel Marthaler, Giampiero Salvi,
Mark van der Wilk, Marco Fraccaro, Dali Wei, Tan Yan Rui, Ernst Kloppenburg, Ryan Turner, Seth Flaxman and Chris Williams.
Especially Ed Snelson helped to improve the code and to include sparse approximations and Roman Garnett and
José Vallet helped to include hyperparameter priors. The spectral mixture covariance function and the grid-based
inference were contributed by Andrew Gordon Wilson and periodic covariances were added by James Robert Lloyd. The improved
variational KL implementation comes from Emtiyaz Khan and Wu Lin and the Ornstein-Uhlenbeck and Langevin covariance functions
are by Juan Pablo Carbajal and Robert MacKay, respectively. Improved logdet estimation for grid-based covariance approximations
were contributed by Kun Dong and Insu Han.
State space inference code was developed together with Arno Solin and Alex Grigorievskiy.

---

Last modified: March 20th, 2019.
